# MRI vs CT for Baseline Imaging Evaluation in Acute Large Artery Ischemic Stroke: A Subanalysis of the SWIFT-DIRECT Trial

**DOI:** 10.1212/WNL.0000000000207922

**Published:** 2023-12-14

**Authors:** Joachim Fladt, Johannes Kaesmacher, Thomas R. Meinel, Lukas Bütikofer, Daniel Strbian, Omer F. Eker, Jean-Francois Albucher, Hubert Desal, Gaultier Marnat, Chrysanthi Papagiannaki, Sebastien Richard, Manuel Requena, Bertrand Lapergue, Paolo Pagano, Marielle Ernst, Martin Wiesmann, Marion Boulanger, David S. Liebeskind, Jan Gralla, Urs Fischer, and on behalf of the SWIFT DIRECT trial investigators

**Affiliations:** From the Stroke Center and Department of Neurology (J.F., U.F.), University Hospital Basel and University of Basel; University Institute of Diagnostic and Interventional Neuroradiology (J.K., J.G.), and Department of Neurology (T.R.M., U.F.), Inselspital, Bern University Hospital and University of Bern; CTU Bern (L.B.), University of Bern, Switzerland; Department of Neurology (D.S.), Helsinki University Hospital, University of Helsinki, Finland; Department of Neuroradiology (O.F.E.), Hospices Civils de Lyon; Department of Diagnostic and Therapeutic Neuroradiology (J.-F.A.), Centre Hospitalier Universitaire de Toulouse; Department of Diagnostic and Interventional Neuroradiology (H.D.), Centre Hospitalier Universitaire de Nantes, Nantes Université; Interventional and Diagnostic Neuroradiology (G.M.), CHU Bordeaux, University of Bordeaux; Department of Radiology (C.P.), CHU Rouen; Stroke Unit (S.R.), Department of Neurology, CHRU-Nancy, Université de Lorraine, France; Stroke Unit (M.R.), Department of Neurology, Hospital Vall d'Heborn, Barcelona, Spain; Department of Stroke and Diagnostic and Interventional Neuroradiology (B.L.), Foch Hospital, Suresnes, France; Department of Neuroradiology (P.P.), CHU Reims, France; Department of Neuroradiology (M.E.), University Medical Center Goettingen; Department of Neuroradiology (M.W.), University Hospital RWTH Aachen, Germany; Department of Neurology (M.B.), CHU Caen Normandie, University Caen Normandie, INSERM U1237, France; and Department of Neurology and Comprehensive Stroke Center (D.S.L.), David Geffen School of Medicine, University of California, Los Angeles.

## Abstract

**Background and Objectives:**

Whether MRI or CT is preferable for the evaluation of patients with suspected stroke remains a matter of debate, given that the imaging modality acquired at baseline may be a relevant determinant of workflow delays and outcomes with it, in patients with stroke undergoing acute reperfusion therapies.

**Methods:**

In this post hoc analysis of the SWIFT-DIRECT trial that investigated noninferiority of thrombectomy alone vs IV thrombolysis (IVT) + thrombectomy in patients with an acute ischemic anterior circulation large vessel occlusive stroke eligible to receive IVT within 4.5 hours after last seen well, we tested for a potential interaction between baseline imaging modality (MRI/MR-angiography [MRA] vs CT/CT-angiography [CTA]) and the effect of acute treatment (thrombectomy vs IVT + thrombectomy) on clinical and safety outcomes and procedural metrics (primary analysis). Moreover, we examined the association between baseline imaging modality and these outcomes using regression models adjusted for age, sex, baseline NIH Stroke Scale (NIHSS), occlusion location, and Alberta Stroke Program Early CT Score (ASPECTS) (secondary analysis). Endpoints included workflow times, the modified Rankin scale (mRS) score at 90 days, the rate of successful reperfusion, the odds for early neurologic deterioration within 24 hours, and the risk of symptomatic intracranial hemorrhage. The imaging modality acquired was chosen at the discretion of the treating physicians and commonly reflects center-specific standard procedures.

**Results:**

Four hundred five of 408 patients enrolled in the SWIFT-DIRECT trial were included in this substudy. Two hundred (49.4%) patients underwent MRI/MRA, and 205 (50.6%) underwent CT/CTA. Patients with MRI/MRA had lower NIHSS scores (16 [interquartile range (IQR) 12–20] vs 18 [IQR 14–20], *p* = 0.012) and lower ASPECTS (8 [IQR 6–9] vs 8 [IQR 7–9], *p* = 0.021) compared with those with CT/CTA. In terms of the primary analysis, we found no evidence for an interaction between baseline imaging modality and the effect of IVT + thrombectomy vs thrombectomy alone. Regarding the secondary analysis, MRI/MRA acquisition was associated with workflow delays of approximately 20 minutes, higher odds of functional independence at 90 days (adjusted odds ratio [aOR] 1.65, 95% CI 1.07–2.56), and similar mortality rates (aOR 0.73, 95% CI 0.36–1.47) compared with CT/CTA.

**Discussion:**

This post hoc analysis does not suggest treatment effect heterogeneity of IVT + thrombectomy vs thrombectomy alone in large artery stroke patients with different imaging modalities. There was no evidence that functional outcome at 90 days was less favorable following MRI/MRA at baseline compared with CT/CTA, despite significant workflow delays.

**Trial Registration Information:**

ClinicalTrials.gov Identifier: NCT03192332.

## Introduction

According to the current American Heart Association/American Stroke Association Guidelines for the early management of patients with acute ischemic stroke,^1^ both CT/CT-angiography (CTA) and MRI/MR-angiography (MRA) plus optional perfusion imaging may be equally used for baseline imaging evaluation in patients with suspected stroke. Neuroimaging on hospital admission not only enables clinicians to reliably establish the diagnosis of ischemic stroke by ruling out hematoma or tumor and identifying intracranial vessel occlusions but may also allow for the assessment of tissue viability and/or perfusion status, thereby facilitating the selection of suitable candidates for acute treatments.^1^ CT is the most commonly used imaging modality because it is readily available in most stroke care providing centers given the swift image acquisition and optimized in-hospital workflow. Advantages of MRI over CT with those of CT include higher sensitivity in detecting areas of ischemia^2,3^ and superior diagnostic capacity in differentiating stroke from other disorders with stroke-like presentation, such as stroke mimics.^4,5^ However, MRI acquisitions require additional time for both preparations and scanning and may therefore induce workflow slowdowns and delayed treatment, thus potentially resulting in poorer outcomes.^6^ The most recent comparisons of MRI and CT acquisition for acute stroke evaluation in the literature have not revealed significant differences between clinical outcomes by baseline imaging modality, but these studies were based on relatively heterogenous albeit large observational samples prone to bias. Clinical trial populations have the advantage that they are largely standardized, given the strict study enrollment criteria.

In this subanalysis of the SWIFT-DIRECT trial, we sought to assess how the choice of imaging modality (MRI/MRA vs CT/CTA) used for stroke evaluation on hospital admission influences procedural metrics and outcomes after acute stroke therapy in a clinical trial setting. To this end, we (1) tested for a potential interaction between baseline imaging modality (MRI/MRA vs CT/CTA) and the effect of IV thrombolysis (IVT) + thrombectomy vs thrombectomy alone on clinical and safety outcomes and procedural metrics and (2) examined the impact of baseline imaging modality on these outcomes.

## Methods

### Standard Protocol Approvals, Registrations, and Patient Consents

This was a post hoc subanalysis of the SWIFT-DIRECT trial (ClinicalTrials.gov Identifier: NCT03192332). We report all findings of this subanalysis following the Consolidated Standards of Reporting Trials (CONSORT) guidelines. Written informed consent was provided by all study participants or their legally authorized representatives. The study was approved by the ethics board and the local authorities at each participating center.

### Study Design and Patient Sample

SWIFT-DIRECT was a multicenter, international, randomized, open-label, blinded endpoint trial that investigated whether thrombectomy alone was noninferior to IVT + thrombectomy in patients with an acute ischemic anterior circulation large vessel occlusive stroke eligible to receive IVT within 4.5 hours after last seen well presenting directly to a thrombectomy-capable stroke center. The main results^7^ and the trial protocol^8^ have been previously published. To establish eligibility for study enrollment, patients with suspected stroke were screened on admission based on a clinical assessment (anamnesis plus neurologic examination), to determine a relevant neurologic deficit, and baseline imaging, to constitute the diagnosis of an acute ischemic stroke amenable to thrombectomy and IVT, before included patients were randomly assigned (1:1 ratio) to receive thrombectomy alone or IVT + thrombectomy (IV alteplase, 0.9 mg/kg body weight, maximum dose 90 mg) using a centralized web server. In brief, inclusion criteria were (1) occlusion of the intracranial carotid artery (ICA) and/or the first segment (M1) of the middle cerebral artery, confirmed on CTA or MRA; (2) eligibility to receive IV alteplase within 4.5 hours after last seen well and able to undergo thrombectomy within 75 minutes of randomization; (3) severe neurologic deficits, defined as an NIH Stroke Scale (NIHSS) score of ≥5. Patients were not eligible if they had advanced dementia, significant premorbid disabilities, or severe early tissue injury (Alberta Stroke Program Early CT Score [ASPECTS] <5) on baseline noncontrast CT or diffusion-weighted MRI. On MRI, ASPECTS regions had to exhibit at least 20% diffusion abnormality to be considered “positive” on MR-ASPECTS.^8^

The modality of baseline imaging was not specified in the study protocol. For patients included in this substudy, baseline imaging had to involve either CT/CTA or MRI/MRA only. The choice of the imaging modality was left to the discretion of the treating physicians and usually reflects standard operating procedures of participating centers. Patients who underwent more than one imaging modality on admission were excluded. Detailed information on MRI protocols and scanner specifications are summarized in eTable 1 (links.lww.com/WNL/D277) for centers that performed baseline MRI at least in a subset of their enrolled patients. As a sensitivity analysis, ASPECTS were increased by 1 point in patients who underwent MRI evaluation, based on the notion that ASPECTS assessment on MRI tends to be more sensitive and can lead to lower scores.^9^

### Outcomes

The primary outcome was functional independence at 90 days, defined as modified Rankin scale (mRS) score ≤2. Secondary outcomes included the mRS score shift on an ordinal scale and all-cause mortality at 90 days, a neurologic deterioration defined as a ≥4-point increase in the NIHSS score at 24 hours, achieved final cross sectional expanded treatment in cerebral infarction score (cs-eTICI) ≥2b, asymptomatic intracranial hemorrhage (aICH; any parenchymal hematoma or hemorrhage that is not associated with a ≥4-point increase in the NIHSS at 24 hours) and symptomatic intracranial hemorrhage (sICH; i.e., associated with a ≥4-point increase in the NIHSS score within 24 hours), time from arrival to groin puncture (door-to-puncture time), time from arrival to successful reperfusion on the final angiography run, cs-eTICI ≥2b (door-to-reperfusion time), and the time from arrival to alteplase bolus (door-to-needle time; only in IVT + thrombectomy group).

### Statistical Analysis

All analyses were performed by an independent statistician (L.B.) according to a prespecified statistical analysis plan (available in supplemental material). Patient characteristics stratified by baseline imaging modality (MRI vs CT) were summarized using descriptive statistics and reported as counts/percentages for categorical variables and medians/interquartile ranges (IQRs) for continuous variables. For statistical comparisons, the Fisher exact test was used for categorical variables and the Mann-Whitney Wilcoxon test for continuous and ordinal variables. For the primary analysis, we assessed the interaction between allocation to IVT + thrombectomy vs thrombectomy and baseline imaging modality using regression models appropriate to the respective outcome with baseline imaging modality, allocation, and their interaction as covariates. Marginal effects in each treatment group and the *p* value for the interaction term were reported. For the secondary analysis, we used similar regression models with baseline imaging modality as covariate. Binary outcomes were analyzed using Firth logistic regression,^10^ a penalized maximum likelihood method that reduces small-sample bias (a); shift in mRS score was analyzed using ordinal logistic regression (b); and time outcomes were analyzed using linear regression with robust standard errors (c). Effects are reported as odds ratio (OR) with 95% CI (a), OR for better outcome (lower mRS score) with 95% CI (b), and mean difference (MD) with 95% CI (c). All regression models were adjusted for sex and the binary stratification variables from randomization in the main study, that is, NIHSS at baseline (≤17 vs >17), age (<70 vs ≥70 years), occlusion location (M1 only vs Intracranial ICA or Intracranial ICA and M1), and ASPECTS (4–7 vs 8–10). Statistical tests were 2-sided. All analyses were conducted using Stata/MP 17.0 (StataCorp, College Station, TX).

### Data Availability

The anonymized SWIFT-DIRECT dataset will be available on reasonable request to the corresponding author.

## Results

### Cohort Characteristics

Four hundred five of 408 patients enrolled in the SWIFT-DIRECT trial met the inclusion criteria for this substudy, while 3 patients were excluded because they had undergone both imaging modalities (CT and MRI) on admission. Data were >95% complete for all outcomes (CONSORT flowchart eFigure 1, links.lww.com/WNL/D276). The median age was 72 years (IQR 64–81), and the cohort comprised 209 (51.6%) women. Overall, patients arrived at the hospital at a median of 75 minutes (IQR 56–108) after stroke onset. The median NIHSS in the overall cohort was 17 (IQR 13–20). Baseline imaging was acquired using MRI in 200 patients (49.4%) and CT in 205 patients (50.6%). For the assessment of tissue at risk, 330 patients (81.5%) underwent advanced imaging (i.e., MRI or CT perfusion) on admission and 75 patients (18.5%) received basic imaging (i.e., CT/CTA). Overall, the door-to-puncture time was 77 minutes (IQR 62–94) and the door-to-reperfusion time was 114 minutes (IQR 96–138) in patients with successful reperfusion (n = 366, 93.1%). Baseline characteristics of the cohort are summarized in Table 1.

**Table 1:** Cohort Characteristics

	Total (N = 405)		CT (N = 205)		MRI (N = 200)		*p* Value
	N^a^		N^a^		N^a^		
Group, n (%)	405		205		200		0.49
Thrombectomy alone		200 (49.4)		105 (51.2)		95 (47.5)	
Alteplase plus thrombectomy		205 (50.6)		100 (48.8)		105 (52.5)	
Age at inclusion, median (IQR)	405	72 (64–81)	205	73 (64–81)	200	72 (63–81)	0.40
Female sex, n (%)	405	209 (51.6)	205	104 (50.7)	200	105 (52.5)	0.77
NIHSS, median (IQR)	405	17 (13–20)	205	18 (14–20)	200	16 (12–20)	0.012
Prestroke mRS, n (%)	405		205		200		1.00
0		343 (84.7)		173 (84.4)		170 (85.0)	
1		61 (15.1)		31 (15.1)		30 (15.0)	
4		1 (0.2)		1 (0.5)		0 (0.0)	
Weight (kg), median (IQR)	379	75 (65–85)	187	75 (65–85)	192	75 (65–85)	0.86
Systolic blood pressure (mm Hg), median (IQR)	400	147 (131–162)	203	150 (138–167)	197	144 (129–160)	0.002
Diastolic blood pressure (mm Hg), median (IQR)	397	80 (70–90)	201	82 (71–90)	196	79 (70–90)	0.08
Heart rate (beats per minute), median (IQR)	394	74 (64–87)	201	76 (64–88)	193	73 (63–86)	0.60
Risk factors							
Previous ischemic stroke, n (%)	391	40 (10.2)	194	18 (9.3)	197	22 (11.2)	0.62
Previous transient ischemic attack, n (%)	386	21 (5.4)	192	10 (5.2)	194	11 (5.7)	1.00
History of hypertension, n (%)	395	237 (60.0)	198	120 (60.6)	197	117 (59.4)	0.84
History of atrial fibrillation, n (%)	384	38 (9.9)	190	21 (11.1)	194	17 (8.8)	0.50
History of hypercholesterolemia, n (%)	384	129 (33.6)	190	67 (35.3)	194	62 (32.0)	0.52
Previous intracerebral hemorrhage, n (%)	394	2 (0.5)	198	1 (0.5)	196	1 (0.5)	1.00
Prior myocardial infarction, n (%)	387	40 (10.3)	192	21 (10.9)	195	19 (9.7)	0.74
Medication							
Warfarin or other anticoagulant, n (%)	405	15 (3.7)	205	11 (5.4)	200	4 (2.0)	0.11
Aspirin, n (%)	405	103 (25.4)	205	47 (22.9)	200	56 (28.0)	0.26
Statine or other lipid-lowering agent, n (%)	405	117 (28.9)	205	54 (26.3)	200	63 (31.5)	0.27

Laboratory values	Total (N = 405)		CT (N = 205)		MRI (N = 200)		*p* Value
	N^a^		N^a^		N^a^		
Blood glucose level (mmol/L), median (IQR)	383	6.5 (5.8–7.5)	198	6.4 (5.8–7.5)	185	6.6 (5.8–7.7)	0.46
International normalized ratio (INR), median (IQR)	318	1.0 (1.0–1.1)	174	1.0 (1.0–1.1)	144	1.0 (1.0–1.1)	0.98
Platelet count ×10^9^ (g/L), median (IQR)	402	226 (189–272)	205	225 (188–268)	197	226 (189–274)	0.73
Hemoglobin (g/L), median (IQR)	405	137 (125–147)	205	138 (123–147)	200	137 (127–145)	0.96
Glomerular filtration rate (mL/min), median (IQR)	405	76 (61–90)	205	74 (61–90)	200	78 (62–90)	0.39
ASPECTS (core laboratory), median (IQR)	404	8.0 (7.0–9.0)	204	8.0 (7.0–9.0)	200	8.0 (6.0–9.0)	0.021
ASPECTS (corrected), median (IQR)	404	8.0 (7.0–9.0)	204	8.0 (7.0–9.0)	200	9.0 (7.0–10)	<0.001
Baseline intracranial occlusion site, n (%)	405		205		200		0.53
Distal ICA-I		16 (4.0)		10 (4.9)		6 (3.0)	
Distal ICA-I and M1		2 (0.5)		2 (1.0)		0 (0.0)	
Distal ICA-L		54 (13.3)		29 (14.1)		25 (12.5)	
Distal ICA-T		44 (10.9)		23 (11.2)		21 (10.5)	
Distal M1		125 (30.9)		66 (32.2)		59 (29.5)	
Distal M2		4 (1.0)		3 (1.5)		1 (0.5)	
Proximal M1		142 (35.1)		65 (31.7)		77 (38.5)	
Proximal M2		18 (4.4)		7 (3.4)		11 (5.5)	
Distal occlusion sites,^b^ n (%)	405		205		200		0.76
No		258 (63.7)		129 (62.9)		129 (64.5)	
Yes		147 (36.3)		76 (37.1)		71 (35.5)	
Tandem lesion, n (%)	405	63 (15.6)	205	38 (18.5)	200	25 (12.5)	0.10
Stroke etiology, n (%)	405		205		200		0.06
Large artery atherosclerosis		68 (16.8)		36 (17.6)		32 (16.0)	
Cardioembolism		154 (38.0)		75 (36.6)		79 (39.5)	
Other determined etiology		18 (4.4)		4 (2.0)		14 (7.0)	
Undetermined etiology		165 (40.7)		90 (43.9)		75 (37.5)	
Time from stroke onset to hospital arrival (min), median (IQR)	405	75 (56–108)	205	70 (50–99)	200	85 (62–120)	<0.001
Time from stroke onset to randomization (min), median (IQR)	405	129 (100–170)	205	113 (83–141)	200	145 (118–182)	<0.001
Time from randomization to groin puncture (min), median (IQR)	405	28 (20–38)	205	27 (20–38)	200	29 (20–38)	0.82

Abbreviations: ASPECTS = Alberta Stroke Program Early CT Score; ICA = internal carotid artery; ICH = intracranial hemorrhage; IQR = interquartile range; MCA = middle cerebral artery; mRS = modified Rankin scale; NIHSS = NIH Stroke Scale.

Baseline characteristics are reported as counts/percentages for categorical variables and medians/IQRs for continuous variables.

a N: number of patients with nonmissing data.

b Distal occlusion sites refer to the A2 segment of the anterior cerebral artery or M3, M4 segments of MCA.

Patients with MRI at baseline had lower NIHSS scores (16 [IQR 12–20] vs 18 [IQR 14–20], *p* = 0.012) and lower ASPECTS (8 [IQR 6–9] vs 8 [IQR 7–9], *p* = 0.021) compared with patients with CT/CTA. The median systolic blood pressure was lower in the MRI group compared with that in the CT group (144 mm Hg [IQR 129–160] vs 150 mm Hg [IQR 138–167]). We found no evidence of imbalances in other patient characteristics (Table 1).

### Primary Analysis: Effect of Allocation to IVT + Thrombectomy vs Thrombectomy by Baseline Imaging Modality (MRI vs CT)

Overall, patients who received IVT + thrombectomy had greater odds of being independent at 90 days compared with those who underwent thrombectomy alone (adjusted OR [aOR] 1.53, 95% CI 1.00–2.34, *p* = 0.048). There was no differential effect (*p* interaction = 0.45) between patients evaluated with CT (aOR 1.78, 95% CI 0.98–3.23, *p* = 0.06) and patients undergoing baseline MRI (aOR 1.28, 95% CI 0.69–2.36, *p* = 0.44). Successful reperfusion (cs-eTICI 2c/3) was more often achieved after IVT + thrombectomy than thrombectomy alone in patients with baseline CT (aOR 3.40, 95% CI 1.05–10.98, *p* = 0.041), while in those undergoing MRI, cs-eTICI 2c/3 was found similarly frequent after IVT + thrombectomy and thrombectomy alone (aOR 1.57, 95% CI 0.45–5.45, *p* = 0.47). The effect of IVT + thrombectomy vs thrombectomy alone on the reperfusion grade was not modified by the choice of baseline imaging modality (*p* interaction = 0.38). Mortality was similar after IVT + thrombectomy and thrombectomy alone (aOR 0.61, 95% CI 0.30–1.23, *p* = 0.17) both in patients with CT (aOR 0.48, 95% CI 0.19–1.23, *p* = 0.13) and MRI (aOR 0.78, 95% CI 0.28–2.20, *p* = 0.64) (*p* interaction = 0.50). For the remaining primary or secondary outcomes, there was no evidence for an interaction between the effect of allocation to IVT + thrombectomy vs thrombectomy alone and the imaging modality used at baseline after adjusting for potential confounders (Table 2).

**Table 2:** Effect of Allocation to Alteplase Plus Thrombectomy (MT + IVT) vs Thrombectomy Alone (MT) on All Outcomes by Baseline Imaging Modality (MRI vs CT)

	N	MT, n/N (%) or median (IQR)	MT + IVT, n/N (%) or median (IQR)	OR (95% CI)	*p* Value	*p* Value for interaction
mRS 0–2 at 90 d	404	112/199 (56.3)	134/205 (65.4)	1.53 (1.00–2.34)	0.048	
CT	204	54/104 (51.9)	61/100 (61.0)	1.78 (0.98–3.23)	0.06	0.45
MRI	200	58/95 (61.1)	73/105 (69.5)	1.28 (0.69–2.36)	0.44	
mRS at 90 d	404	2.0 (1.0–4.0)	2.0 (1.0–3.0)	1.39 (0.98–1.96)	0.07	
CT	204	2.0 (1.0–4.0)	2.0 (1.0–3.0)	1.81 (1.06–3.11)	0.031	0.14
MRI	200	2.0 (1.0–4.0)	2.0 (1.0–3.0)	1.05 (0.66–1.69)	0.83	
NIHSS increase ≥4 at 24 h	397	19/196 (9.7)	17/201 (8.5)	0.87 (0.44–1.74)	0.69	
CT	201	11/103 (10.7)	14/98 (14.3)	1.16 (0.49–2.75)	0.74	0.20
MRI	196	8/93 (8.6)	3/103 (2.9)	0.42 (0.11–1.52)	0.18	
Post cs-eTICI 2bc/3	393	176/195 (90.3)	190/198 (96.0)	2.45 (1.04–5.74)	0.040	
CT	194	88/100 (88.0)	90/94 (95.7)	3.40 (1.05–10.98)	0.041	0.38
MRI	199	88/95 (92.6)	100/104 (96.2)	1.57 (0.45–5.45)	0.47	
Mortality at 90 d	404	23/199 (11.6)	16/205 (7.8)	0.61 (0.30–1.23)	0.17	
CT	204	13/104 (12.5)	9/100 (9.0)	0.48 (0.19–1.23)	0.13	0.50
MRI	200	10/95 (10.5)	7/105 (6.7)	0.78 (0.28–2.20)	0.64	
Hemorrhage at 24 h	404	58/200 (29.0)	69/204 (33.8)	1.24 (0.81–1.90)	0.32	
CT	204	32/105 (30.5)	32/99 (32.3)	1.00 (0.55–1.82)	0.99	0.32
MRI	200	26/95 (27.4)	37/105 (35.2)	1.55 (0.84–2.85)	0.16	
Symptomatic ICH at 24 h	401	5/200 (2.5)	7/201 (3.5)	1.37 (0.45–4.19)	0.58	
CT	203	5/105 (4.8)	6/98 (6.1)	1.23 (0.37–4.07)	0.73	0.61
MRI	198	0/95 (0.0)	1/103 (1.0)	2.98 (0.12–74.58)	0.51	
Asymptomatic ICH at 24 h	401	13/200 (6.5)	14/201 (7.0)	1.08 (0.50–2.32)	0.84	
CT	203	10/105 (9.5)	7/98 (7.1)	0.73 (0.27–1.94)	0.53	0.20
MRI	198	3/95 (3.2)	7/103 (6.8)	2.13 (0.58–7.86)	0.26	
		**MT, median (IQR)**	**MT + IVT, median (IQR)**	**MD (95% CI)**		
Door-to-puncture time (min)	405	75.0 (60.0–90.0)	80.0 (63.0–101.0)	5.34 (0.57 to 10.12)	0.028	
CT	205	65.0 (52.0–82.0)	67.5 (53.0–85.0)	2.19 (−4.57 to 8.95)	0.52	0.27
MRI	200	81.0 (70.0–94.0)	87.0 (75.0–106.0)	7.32 (1.32 to 13.31)	0.017	
Door-to-reperfusion time (min)	355	110.0 (91.0–136.0)	118.0 (99.0–139.5)	2.89 (−4.85 to 10.63)	0.46	
CT	175	102.0 (87.0–125.0)	114.0 (91.0–140.0)	7.04 (−3.40 to 17.47)	0.19	0.28
MRI	180	116.0 (100.0–149.0)	120.0 (106.0–139.0)	−1.28 (−12.18 to 9.62)	0.82	

Abbreviations: cs-eTICI = cross sectional expanded treatment in cerebral infarction score; ICH = intracranial hemorrhage; IQR = interquartile range; IVT = IV thrombolysis; MD = mean difference; mRS = modified Rankin scale; MT = mechanical thrombectomy; NIHSS = NIH Stroke Scale; OR = odds ratio.

Effects are provided as marginal OR or MD with 95% CI based on firth logistic, ordinal logistic, or linear regression models adjusted for stratification factors and sex.

### Secondary Analysis: Association of Baseline Imaging Modality (MRI vs CT) With Outcomes

**Figure 1:**
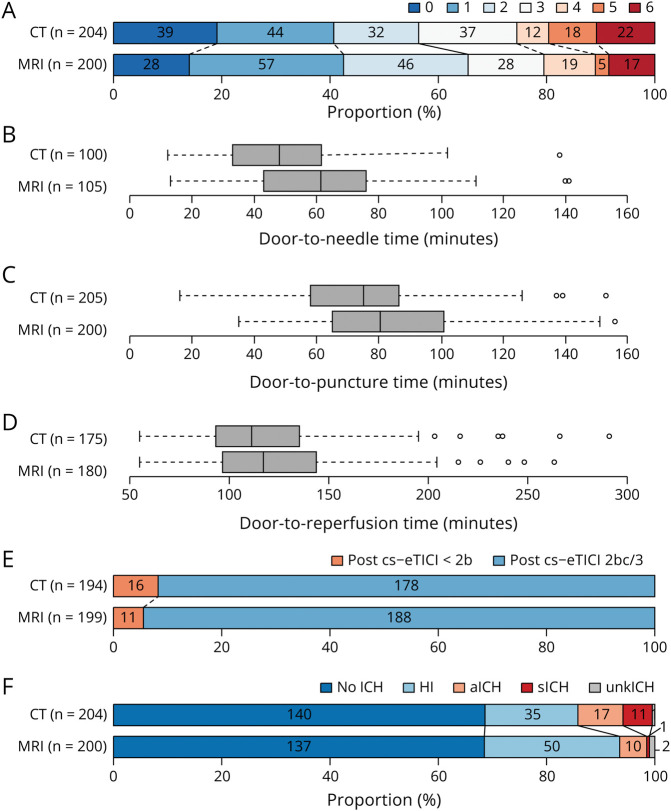
Adjusted Clinical and Procedural Outcome Distribution and Workflow Metrics by Baseline Imaging Modality (CT vs MRI) Descriptive primary and secondary outcomes by baseline imaging (MRI vs CT). (A) mRS at the 90-day visit, (B) door-to-needle time for patients receiving IVT, (C) door-to-puncture time, (D) door-to-reperfusion if cs-eTICI 2b/3, (E) postinterventional reperfusion (cs-eTICI 2b/3), (F) ICH, separated in hemorrhagic infarction, aICH, sICH, and unkICH. aICH = asymptomatic ICH; eTICI = expanded treatment in cerebral infarction score; HI = hemorrhagic infarction; ICH = intracranial hemorrhage; mRS = modified Rankin scale; sICH = symptomatic ICH; unkICH = ICH with unknown symptomatology.

Baseline imaging using MRI was associated with higher chances of being functionally independent (mRS ≤2) at 90 days (aOR 1.65, 95% CI 1.07–2.56) after adjustment for potential confounders (Table 3, Figure 1). Patients evaluated with MRI at baseline were less likely to experience a ≥4-point increase in the NIHSS at 24 hours (aOR 0.33, 95% CI 0.15–0.71) and had a lower risk to experience sICH (aOR 0.10, 95% CI 0.02–0.54). No differences between the MRI and the CT group were found regarding the chances of achieving successful reperfusion after thrombectomy (aOR 1.91, 95% CI 0.83–4.36), the risk of aICH (aOR 0.64, 95% CI 0.29–1.43), and mortality at 90 days (aOR 0.73, 95% CI 0.36–1.47). MRI acquisition at baseline was associated with a longer median door-to-puncture time (MD 16.6 minutes, 95% CI 12.0–21.3) and a longer door-to-reperfusion time in those with successful reperfusion (MD 15.7 minutes, 95% CI 7.6–23.7), compared with CT. In the subgroup of patients randomized to IVT + thrombectomy, MRI acquisition at baseline was associated with a longer door-to-needle time compared with CT (MD 20.9 minutes, 95% CI 14.3–27.5) (Table 3).

**Table 3:** Association of Baseline Imaging Modality (MRI vs CT) With All Outcomes, Adjusted for Core Laboratory ASPECTS (≤7 vs >7), the Other Stratification Factors, and Sex

	N	CT, n/N (%) or median (IQR)	MRI, n/N (%) or median (IQR)	OR (95% CI)	*p* Value
mRS 0–2 at 90 d	404	115/204 (56.4)	131/200 (65.5)	1.65 (1.07–2.56)	0.024
mRS at 90 d	404	2.0 (1.0–4.0)	2.0 (1.0–3.0)	1.28 (0.90–1.83)	0.17
NIHSS increase ≥4 at 24 h	397	25/201 (12.4)	11/196 (5.6)	0.33 (0.15–0.71)	0.004
Post cs-eTICI 2bc/3	393	178/194 (91.8)	188/199 (94.5)	1.91 (0.83–4.36)	0.13
Mortality at 90 d	404	22/204 (10.8)	17/200 (8.5)	0.73 (0.36–1.47)	0.38
Hemorrhage at 24 h	404	64/204 (31.4)	63/200 (31.5)	0.97 (0.63–1.50)	0.90
Symptomatic ICH at 24 h	401	11/203 (5.4)	1/198 (0.5)	0.10 (0.02–0.54)	0.008
Asymptomatic ICH at 24 h	401	17/203 (8.4)	10/198 (5.1)	0.64 (0.29–1.43)	0.28
		**CT, median (IQR)**	**MRI, median (IQR)**	**MD (95% CI)**	
Door-to-needle time (min)	205	41.0 (31.0–58.5)	65.0 (50.0–80.0)	20.9 (14.3–27.5)	<0.001
Door-to-puncture time (min)	405	66.0 (52.0–85.0)	85.0 (73.0–102.0)	16.6 (12.0–21.3)	<0.001
Door-to-reperfusion time (min)	355	106.0 (87.0–135.0)	118.0 (104.0–144.5)	15.7 (7.6–23.7)	<0.001
Imaging-to-puncture time (min)	370	51.0 (39.0–63.0)	61.0 (52.5–75.0)	12.3 (8.3–16.3)	<0.001

Abbreviations: ASPECTS = Alberta Stroke Program Early CT Score; cs-eTICI = cross sectional expanded treatment in cerebral infarction score; ICH = intracranial hemorrhage; IQR = interquartile range; MD = mean difference; mRS = modified Rankin scale; NIHSS = NIH Stroke Scale; OR = odds ratio.

Effects are provided as OR (with the CT group being the reference) or MD with 95% CI, based on firth logistic, ordinal logistic, or linear regression models adjusted for stratification factors and sex.

### Sensitivity Analysis: “Corrected” ASPECTS

When using corrected ASPECTS, that is, a 1-point increase in ASPECTS if assessment had been performed on MRI, no differences between patients with baseline MRI and those with CT were found in terms of the mRS scores on an ordinal scale at 90 days (aOR 1.13, 95% CI 0.80–1.61) and for the rates of functional independence (mRS ≤2) at 90 days (aOR 1.45, 95% CI 0.95–2.22), successful reperfusion after thrombectomy (aOR 1.54, 95% CI 0.69–3.46), aICH (aOR 0.64, 95% CI 0.29–1.41), and mortality (aOR 0.85, 95% CI 0.42–1.69). Patients undergoing MRI at baseline were less likely to experience a ≥4-point increase in the NIHSS at 24 hours (aOR 0.41, 95% CI 0.19–0.88) or sICH (aOR 0.13, 95% CI 0.02–0.70) (eTable 2, links.lww.com/WNL/D278).

## Discussion

In this substudy of the SWIFT-DIRECT trial^7^ with a well-balanced proportion of patients who underwent MRI for baseline stroke evaluation (49.4%), we obtained the following main findings: (1) Baseline imaging modality did not modify the effect of IVT + thrombectomy vs thrombectomy alone on conventional stroke outcome parameters. (2) Patients undergoing MRI on admission were subject to measurable treatment delays, that is, they had significantly longer door-to-needle and door-to-puncture times compared with patients receiving CT, and reperfusion was achieved later in the MRI group. (3) Rates of successful reperfusion in both groups were similar, while patients with MRI had lower odds of early complications, such as a relevant NIHSS increase or sICH within 24 hours, compared with patients undergoing CT. (4) Compatibly, patients triaged with MRI at baseline were more likely to achieve functional independence and showed similar mortality at 90 days compared with patients with CT.

This substudy remains inconclusive in terms of the primary aim, that is, potential treatment effect heterogeneity of IVT + thrombectomy vs thrombectomy alone across different baseline imaging modalities. We did not show a statistical interaction suggesting no significant advantage of one or the other imaging modality in terms of the effect of aforementioned treatment allocations tested in the SWIFT-DIRECT trial. However, our study may have lacked sufficient statistical power to answer this question with certainty. Future studies and analyses of pooled trial data could help determine a differential effect of IVT + thrombectomy vs thrombectomy alone that may depend on baseline imaging modality.

Whether MRI or CT is the preferable imaging technique for acute stroke evaluation remains a matter of debate. Several previous studies have reported prolonged imaging to treatment times in patients undergoing MRI for baseline stroke evaluation compared with patients receiving CT.^11-14^ Given the time-dependent effect of both IVT^15^ and thrombectomy^16^ and that short workflow times are thus crucial for acute treatment selection, the additional time requirement of image acquisitions is a major limitation of MRI compared with CT in terms of both main imaging goals, establishing the diagnosis of stroke, and selecting suitable candidates for acute treatments such as IVT and thrombectomy.^17^

Regarding the secondary aim, baseline imaging evaluation using MRI compared with CT was associated with significant delays in the median door-to-puncture time (MD 16.6 minutes, 95% CI 12.0–21.3, *p* < 0.001) in the overall cohort and a longer median door-to-needle time (MD 20.9 minutes, 95% CI 14.3–27.5, *p* < 0.001) in the group allocated to IVT + thrombectomy. The finding of prolonged door-to-needle times after MRI is consistent with reports from previous literature^11-13^ including a recent analysis from the Austrian Stroke Registry.^14^ Of interest, a subanalysis of patients from the THRACE trial^18^ found shorter times from the start of imaging acquisition to initiation of IVT after using MRI for baseline assessment compared with CT, despite longer scan duration in the MRI group.^19^ The authors speculate that treatment decisions may be possible in some cases before MRI acquisition is fully completed, given that signs of ischemia are often more conclusive than on CT where early ischemic changes may be subtle. However, in this study, the time from image acquisition start to groin puncture was longer in patients undergoing MRI (61 vs 51 minutes, *p* < 0.001). This discrepancy is somewhat unexpected because the data of both studies are collected in a clinical trial setting involving predominantly academic centers with optimized treatment workflow.

In terms of door-to-puncture times, findings in literature have been largely heterogeneous. Two analyses of patient cohorts from randomized controlled trials, including the aforementioned THRACE trial,^18^ did not reveal any significant delay of groin puncture in patients undergoing baseline MRI, that is, similar door-to-puncture times in MRI vs CT group.^19,20^ While some studies reporting on real-world observational data have shown similar door-to-puncture times for both modalities,^13^ others found greater delay of the groin puncture in the MRI group.^6,14,21,22^ It has been hypothesized that in-hospital workflows and interdisciplinary communication (e.g., with anesthesia team, neurointerventional technicians) may be more streamlined in academic sites participating in international multicenter trials so that preparatory steps for thrombectomy could partly be initiated in an overlapping manner while completing MR image acquisition. However, the findings of this study seem to contradict such hypothesis and underline that despite continuous efforts on minimizing time requirements of MRI sequences and the implementation of expedited imaging protocols,^23^ workflow delays remain a non-negligible limitation associated with MRI use for acute imaging evaluation.

Despite significant delays in workflow metrics in patients undergoing baseline MRI in this study, clinical outcomes at 90 days tend to favor the MRI group (higher odds of functional independence in MRI vs CT and no difference in mortality risk). This finding is in line with most previous studies on patients treated with IVT^24^ or thrombectomy,^25^ some of which even reported lower mortality for patients undergoing MRI,^26^ while one study found no significant association of MRI-guided IVT/thrombectomy with functional independence or mortality compared with CT.^14^ Several factors may potentially have contributed to this observation. First, using MRI for establishing a stroke diagnosis may allow for consideration of additional imaging features, such as the number of cerebral microbleeds and presence of cortical superficial siderosis on gradient echo sequences sensitive to magnetic susceptibility artifacts or the extent of white matter hyperintensities on T2-weighted MRI, thereby providing a more comprehensive view on patients' overall condition.^27^ This could help establish more individualized risk profiles in terms of early complications after therapy, such as ICH.^28^ For instance, data from this study show a lower risk of sICH in the MRI group compared with that in the CT group (aOR 0.10, 95% CI 0.02–0.54) at similar rates of IV alteplase treatment in both groups (47.5 vs 51.2%). Patients who had undergone MRI on admission were also less likely to experience an early ≥4-point increase in the NIHSS (aOR 0.33, 95% CI 0.15–0.71) than those with CT. Considering similar rates of successful reperfusion after thrombectomy in the MRI and CT groups (aOR 1.91, 95% CI 0.83–4.36), the lower prevalence of early complications may partly explain the more favorable functional outcome in patients with baseline MRI in our study. Second, because using MRI for delineating ischemia and establishing a stroke diagnosis enables more clear-cut treatment indications in most of the cases, borderline imaging profiles—particularly if in combination with challenging clinical scenarios—may discourage some stroke physicians in selecting these patients for acute treatments, such as IVT or thrombectomy, or rather prevent them from enrolling such patients into a trial. This can lead to some extent to overselection of patients with more straightforward clinical and imaging profiles who may be more likely to achieve better functional outcomes. Last and most importantly, because baseline imaging modality was left to the discretion of the treatment team, it is conceivable that patients who were deemed to be in poorer overall condition might have been referred to receive CT in anticipation of potential challenges with MR image acquisition. In fact, patients in this study who received MRI had less severe strokes compared with those undergoing CT (NIHSS 16 [IQR 12–20] vs 18 [IQR 14–20], *p* = 0.012). Of importance, baseline ASPECTS was lower in the MRI group compared with that in the CT group (8 [IQR 6–9] vs 8 [IQR 7–9], *p* = 0.021), which likely represents the higher sensitivity of ASPECTS assessment on MRI leading to lower scores.^9^ This underlines that our data may be subject to assignment bias representing a general limitation of previous studies comparing patient data that have not been randomized by imaging modality. In this regard, results from the ongoing IMAGECAT randomized controlled trial (ClinicalTrials.gov Identifier: NCT03745391) are expected to be instrumental for determining the optimal imaging modality (multimodal MRI or multimodal CT) for baseline stroke evaluation and thrombectomy selection.

Some limitations require consideration. First, patients included in this substudy of the SWIFT-DIRECT trial were not randomized according to the objective of this study, that is, by baseline imaging modality. Hence, potential indication bias in terms of the assignment to either of the study groups (MRI vs CT) is inevitable. However, because baseline MRI was mostly acquired in centers specialized in acute MR imaging evaluation of stroke, choice of MRI by default may rather reflect site policy and be less dependent on the patients' condition except for a small minority of most severely ill patients who are likely to undergo CT. Yet, we cannot exclude that MRI may have identified a set of patients more likely to have better outcome. Second, ASPECTS assessments on MRI—due to higher sensitivity of MRI to ischemic changes—may have resulted in more favorable tissue profiles in patients with baseline MRI, potentially confounding clinical outcome. Accordingly, in a sensitivity analysis using a “corrected” ASPECTS involving a compensatory 1-point increase for MRI patients, baseline MRI evaluation did not remain significantly associated with functional independence. Third, given the strict inclusion criteria (imaging features, premorbid disability status, etc.) of the main study, this patient cohort represents a highly selected sample of patients with relatively favorable stroke profiles that may not be generalizable to the total population of patients with large vessel occlusive stroke. Fourth, this study was not specifically designed to detect an interaction between the effect of assignment to the IVT + thrombectomy group and baseline imaging modality. Therefore, it might be underpowered regarding this objective, and consequently, the absence of any statistically significant interaction in our dataset does not fully exclude a potential effect modification by the choice of imaging technique. Finally, in terms of stroke treatments in the main study, systemic thrombolysis in the IVT + thrombectomy group was restricted to IV alteplase excluding other options, such as tenecteplase. For thrombectomy, use of a Solitaire stent retriever revascularization device from Medtronic was mandated as the first choice. The reported findings may, therefore, not apply to other drugs/devices.

In conclusion, this study does not suggest treatment effect heterogeneity of IVT + thrombectomy vs thrombectomy alone in large artery stroke patients with different imaging modalities. Despite significant in-hospital workflow delays in patients who underwent MRI for baseline stroke evaluation compared with those who underwent CT, increased workflow times associated with MRI acquisition did not have a significant negative impact on overall patient outcomes. Randomized controlled trials are required to clarify whether the tissue-based benefits of MRI compensate for temporal benefits of CT in patients with large artery ischemic stroke.

## Appendix Authors

**Table:**

Name	Location	Contribution
Joachim Fladt, MD	Stroke Center and Department of Neurology, University Hospital Basel and University of Basel, Switzerland	Drafting/revision of the article for content, including medical writing for content; major role in the acquisition of data; study concept or design; and analysis or interpretation of data
Johannes Kaesmacher, MD	University Institute of Diagnostic and Interventional Neuroradiology, Inselspital, Bern University Hospital, and University of Bern, Switzerland	Drafting/revision of the article for content, including medical writing for content; major role in the acquisition of data; study concept or design; and analysis or interpretation of data
Thomas R. Meinel	Department of Neurology, Inselspital, Bern University Hospital, and University of Bern, Switzerland	Drafting/revision of the article for content, including medical writing for content
Lukas Bütikofer	CTU Bern, University of Bern, Switzerland	Drafting/revision of the article for content, including medical writing for content; study concept or design; and analysis or interpretation of data
Daniel Strbian	Department of Neurology, Helsinki University Hospital, University of Helsinki, Finland	Drafting/revision of the article for content, including medical writing for content
Omer F. Eker, MD, PhD	Department of Neuroradiology, Hospices Civils de Lyon, France	Drafting/revision of the article for content, including medical writing for content
Jean-Francois Albucher, MD	Department of Diagnostic and Therapeutic Neuroradiology, Centre Hospitalier Universitaire de Toulouse, France	Drafting/revision of the article for content, including medical writing for content
Hubert Desal	Department of Diagnostic and Interventional Neuroradiology, Centre Hospitalier Universitaire de Nantes, Nantes Université, France	Drafting/revision of the article for content, including medical writing for content
Gaultier Marnat, MD	Interventional and Diagnostic Neuroradiology, CHU Bordeaux, University of Bordeaux, France	Drafting/revision of the article for content, including medical writing for content
Chrysanthi Papagiannaki	Department of Radiology, CHU Rouen, France	Drafting/revision of the article for content, including medical writing for content
Sebastien Richard, PhD	Stroke Unit, Department of Neurology, CHRU-Nancy, Université de Lorraine, France	Drafting/revision of the article for content, including medical writing for content
Manuel Requena, MD, PhD	Stroke Unit, Department of Neurology, Hospital Vall d'Heborn, Barcelona, Spain	Drafting/revision of the article for content, including medical writing for content
Bertrand Lapergue	Department of Stroke and Diagnostic and Interventional Neuroradiology, Foch Hospital, Suresnes, France	Drafting/revision of the article for content, including medical writing for content
Paolo Pagano	Department of Neuroradiology, CHU Reims, France	Drafting/revision of the article for content, including medical writing for content
Marielle Ernst	Department of Neuroradiology, University Medical Center Goettingen, Germany	Drafting/revision of the article for content, including medical writing for content
Martin Wiesmann	Department of Neuroradiology, University Hospital RWTH Aachen, Germany	Drafting/revision of the article for content, including medical writing for content
Marion Boulanger	Department of Neurology, CHU Caen Normandie, University Caen Normandie, INSERM U1237, France	Drafting/revision of the article for content, including medical writing for content
David S. Liebeskind, MD	Department of Neurology and Comprehensive Stroke Center, David Geffen School of Medicine, University of California, Los Angeles	Drafting/revision of the article for content, including medical writing for content
Jan Gralla, MD, MSc	University Institute of Diagnostic and Interventional Neuroradiology, Inselspital, Bern University Hospital, and University of Bern, Switzerland	Drafting/revision of the article for content, including medical writing for content; major role in the acquisition of data; study concept or design; and analysis or interpretation of data
Urs Fischer	Stroke Center and Department of Neurology, University Hospital Basel and University of Basel; Department of Neurology, Inselspital, Bern University Hospital, and University of Bern, Switzerland	Drafting/revision of the article for content, including medical writing for content; major role in the acquisition of data; study concept or design; and analysis or interpretation of data

## Glossary

aICH: asymptomatic ICH
aOR: adjusted odds ratio
ASPECTS: Alberta Stroke Program Early CT Score
CONSORT: Consolidated Standards of Reporting Trials
CTA: CT-angiography
cs-eTICI: cross sectional expanded treatment in cerebral infarction score
ICA: intracranial carotid artery
ICH: intracranial hemorrhage
IQR: interquartile range
MD: mean difference
MRA: MR-angiography
mRS: modified Rankin scale
NIHSS: NIH Stroke Scale
sICH: symptomatic ICH
